# Standard induction with basiliximab versus no induction in low immunological risk kidney transplant recipients: study protocol for a randomized controlled trial

**DOI:** 10.1186/s13063-021-05253-1

**Published:** 2021-06-24

**Authors:** Aziza Ajlan, Hassan Aleid, Tariq Zulfiquar Ali, Hala Joharji, Khalid Almeshari, Ahmed Mohammed Nazmi, Yaser Shah, Edward Devol, Dalal Alkortas, Zinah Alabdulkarim, Dieter Broering, Ibrahim Alahmadi, Asad Ullah, Anwar Alotaibi, Ahmed Aljedai

**Affiliations:** 1 King Faisal Specialist Hospital and Research Centre (KFSHRC), Pharmaceutical Care Division MBC 11, Riyadh, Saudi Arabia; 2 King Faisal Specialist Hospital and Research Centre (KFSHRC), Kidney & Pancreas Health Centre - Riyadh, KPT, Riyadh, Saudi Arabia; 3 Biostats, Epidemiology and Scientific Computing Department, Riyadh, Saudi Arabia; 4 Organ Transplant Centre of Excellence- Riyadh, OTC, Riyadh, Saudi Arabia; 5 Deputyship of Therapeutic Affairs, Ministry of Health, Riyadh, Saudi Arabia

## Abstract

**Background:**

Induction therapy with IL-2 receptor antagonist (IL2-RA) is recommended as a first-line agent in low immunological risk kidney transplant recipients. However, the role of IL2-RA in the setting of tacrolimus-based immunosuppression has not been fully investigated.

**Aims:**

To compare different induction therapeutic strategies with 2 doses of basiliximab vs. no induction in low immunologic risk kidney transplant recipients as per KFSHRC protocol.

**Methods:**

Prospective, randomized, double blind, non-inferiority, controlled clinical trial

**Expected outcomes:**

1. Primary outcomes:

Biopsy-proven acute rejection within first year following transplant

2. Secondary outcomes:

a. Patient and graft survival at 1 year

b. eGFR at 6 months and at 12 months

c. Emergence of de novo donor-specific antibodies (DSAs)

**Trial registration:**

The study has been prospectively registered at clinicaltrials.gov (NTC: 04404127). Registered on 27 May 2020.

**Supplementary Information:**

The online version contains supplementary material available at 10.1186/s13063-021-05253-1.

## Introduction

The standard immunosuppression protocols for solid organ transplantation have evolved to permit reliable short-term graft survival, such that transplantation has become the preferred therapy for end-stage organ failure. In the case of renal transplantation, graft and patient survival exceed 90% during the first post-transplant year [1–3].

Two antibodies have been constructed and produced by recombinant DNA techniques from mouse myeloma cell lines: basiliximab, a chimeric monoclonal antibody which includes the entire murine variable region and is approximately 80% human, and daclizumab, a humanized monoclonal antibody which includes only the hypervariable murine component and is approximately 90% human [4–10]. Only basiliximab is currently on the market with the trade name of Simulect® (basiliximab, Novartis) while Zenapax® (daclizumab, Roche) was withdrawn from the market and have shown virtually the same results in phase III clinical trials, although a randomized, prospective, well-powered study between the two drugs using the currently recommended dosage schedules has not been performed [6, 9, 11–13].

Importantly, the phase II data established that serum basiliximab concentrations exceeding 0.2 μg/ml were sufficient to saturate CD25 on circulating T lymphocytes in vivo [14–16]. Interestingly, the phase II studies were carried out to determine the antibody dose during the first 4 to 6 weeks post-transplant which could achieve this concentration. This concentration can be achieved with single dose basiliximab, i.e., 20 mg [16, 17]. Clinical trials leading to the US FDA drug approval were mainly coupled with cyclosporin as main maintenance immunosuppressant drug. However, the use of basiliximab induction when combined with tacrolimus-based immunosuppression has not been thoroughly investigated in well-structured, randomized clinical trials [18].

### Aims

To compare different induction therapeutic strategies with basiliximab (2 doses vs. no induction, a placebo that is composed of 100 ml of normal saline) administered by primary, registered nurse in low immunologic risk kidney transplant recipients as per KFSHRC protocol (attached).

### Hypothesis

No induction is non-inferior to standard dose induction with basiliximab in low immunological risk kidney transplant recipients.

## Materials and methods

### Study design

**Fig. 1:**
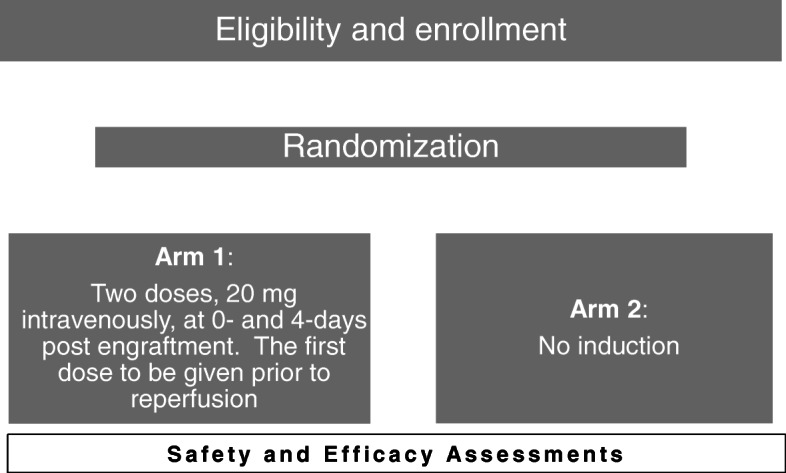
Overview of study procedures

The study is a prospective, randomized, double-blind controlled clinical trial to compare different induction therapeutic strategies in kidney transplant recipients. This study will enroll 70 subjects in each arm. Figure 1 presents a high-level overview of the study procedures. A schedule of assessments is provided in Appendix.

### Study settings

The potential participants will be screened at kidney transplant clinic at King Faisal Specialist Hospital and Research Centre (KFSHRC), Riyadh. KFSHRC is a tertiary referral hospital. It is consistently recognized as one of the nation’s top hospital specializing in Oncology, Organ Transplantation, Cardiovascular Diseases, Neurosciences and Genetic Diseases Potential candidates will be informed of the trial via formal meetings and trial information sheets. This study is reported in line with the Standard Protocol Items; Recommendations for Interventional Trials (SPIRIT) Statement.

### Recruitment

Patients will be recruited from kidney transplant clinic at King Faisal Specialist Hospital and Research Centre (KFSHRC). Involved practitioners will identify potentially suitable patients and, after providing them with information about the study, then will be offered to contact the research team. Upon contact by potential participants, a researcher will explain the study and assess them for study eligibility. If the potential participant remains interested in participating in the study, and after confirming eligibility, a signed consent form will be obtained.

### Study population

In order to be eligible to participate in this study, an individual must meet all the inclusion criteria and none of the exclusion criteria as described below.

#### Inclusion criteria

- Male or female ≥18 years
- Living donor
- Low immunological risk:
  - First (primary) transplant
  - ≤ 4 antigen mismatches (HLA A, B, DR, DQ matching scheme)
  - Negative HLA Ab screening

#### Exclusion criteria

- High immunological risk as per KFSHRC protocol including (see attached protocol for more details Additional file 1)
- HLA identical or zero mismatched transplants
- Receiving cyclosporin as primary maintenance immunosuppressant
- Human immunodeficiency virus (HIV) co-infection
- Pregnant or nursing female
- Has received an investigational medication within the past 30 days
- Has a known contraindication to the administration of basiliximab
- Suspected or known to have a serious infection
- Multi-organ transplant

### Randomization and blinding

After fulfilling the enrolment criteria and obtaining the informed consent, subjects will be randomized to receive either the standard therapy (Arm I) or placebo (no induction) (Arm II) with a 1:1 allocation, as specified in the intervention section below. A randomization algorithm has been generated by the statistician. The randomization algorithm will be maintained by the Department of Pharmacy and has been generated via the ralloc procedure in Stata™ (Jones and Kenward version 3.7.6), with block randomization: subjects will be divided into subgroups called blocks (block size: 8), such that the variability within blocks is less than the variability between blocks. Then, subjects within each block are randomly assigned to either group. The algorithm will be used to randomize patient following signing the informed consent and admission.

#### Blinding

The trial will be blinded with respect to basiliximab induction allocation. The study will be blinded to subjects and site study investigators, with the following exceptions:

- A single pharmacist
- A single biostatistician

Blinding of the basiliximab induction is critical to the integrity of this clinical trial.

In case of an emergency, the principal investigator has the sole responsibility for determining if unblinding of a patient’s treatment assignment is warranted for medical management of the event. The patient safety must always be the first consideration in making such a determination. In other words, in the event of a medical emergency (unexpected serious adverse event) or pregnancy in an individual subject, in which knowledge of the basiliximab induction is deemed critical to the subject’s management, the blind for that subject may be broken by the treating physician.

Before breaking the blind of an individual subject’s blinded treatment, the investigator should have determined that the information is necessary, i.e., that it will alter the subject’s immediate management. The need to break the blind must first be discussed with the study investigators, and the best method to do this will be determined. Un-blinding would be restricted to the specific patient. It is the responsibility of the principal investigator to promptly document the decision and rationale for unblinding a document in the source documents.

#### Pregnancy

Women of childbearing potential (WOCBP) must be using an adequate method of contraception to avoid pregnancy throughout the study and for up to 8 weeks after the study in such a manner that the risk of pregnancy is minimized. WOCBP includes any female who has experienced menarche and who has not undergone successful surgical sterilization (hysterectomy, bilateral tubal ligation, or bilateral oophorectomy) or is not postmenopausal (defined as amenorrhea for 12 consecutive months; or women on hormone replacement therapy [HRT] with documented serum follicle stimulating hormone [FSH] level > 35 mIU/mL). Even women, who are using oral, implanted, or injectable contraceptive hormones or mechanical products such as an intrauterine device or barrier methods (diaphragm, condoms, spermicides) to pre-pregnancy or practicing abstinence or where the partner is sterile (e.g., vasectomy), should be considered to be of childbearing potential. WOCBP must have a negative serum pregnancy test (minimum sensitivity 25 IU/L or equivalent units of human chorionic gonadotropin [HCG]) within 72 h prior to the start of study medication. In case of pregnancy, the participant will withdraw from the study, and management will be differed to primary physician according the standard of care.

### Interim analysis

The patients enrolled in the study will be monitored and interim data analysis will be performed at 6 months and 1 year. This will include: safety, acute rejection, antibody use for rejection, severe rejection, multiple rejections, efficacy failure, cause of graft loss, delayed graft function, study drug discontinuation, renal function and serious adverse events.

### Duration of the study

One year. See Appendix: Schedule of Assessment. However, the patients will be followed as part of standard of care of kidney transplant recipients.

### Statistical methods

The sample size projection is based upon historical data from transplants in 2018 and for which at least 1 year of follow-up has been achieved. These number 16 subjects and from whom one suffered rejection within the year, i.e., a rate of 0.0625. A margin of non-inferiority is proposed such that the true rejection rate among those treated without Simulect® would not be more than 15%, i.e., a margin of 0.0875 (0.1500 minus 0.0625). We recruit patients for the study and calculate a 95% confidence interval for the true rejection rate among those treated without Simulect®. This confidence interval will be P ± SE(p). We wish that the confidence interval for the rate to be at least 0.0875. The standard error of p is SQRT(p(1-p)/n). Solving for *n* yields a value of approximately 64. Accounting for loss to follow-up and other logistical issues, we have added to this number. We propose that the sample size in each arm be 70 subjects.

Statistical analysis will be performed using SAS/JMP Version 14 (SAS Institute, Cary, NC). Baseline parametric data will be expressed as means of and standard deviations and any differences in the groups will be analyzed with the paired Student’s *t* test, the chi-square test, or the Mann-Whitney *U* test as appropriate. *P* values less than 0.05 will be considered statistically significant. Multivariate analysis will be used to adjust for other risk factors including the concomitant administration of other interacting medications such immunosuppressants. Kaplan-Meier survival analyses will be used to compare outcomes for patient survival, graft survival, rejection, and BK nephropathy. Univariate and multivariable stepwise. Cox regression analyses will be performed to determine risk factors associated with patient death, graft loss, and rejection. We will also use propensity score analyses as an additional means to account for group differences in baseline characteristics. A propensity score represents the likelihood of a patient receiving a particular treatment or, in this case, no basiliximab induction instead of two doses of basiliximab. Data analysis will be carried out after completion of therapy in retrospective manner.

## Treatment (interventions)

- *Arm 1*: Two doses of basiliximab, 20 mg intravenously, at 0 and 4 days post engraftment. The first dose to be given prior to reperfusion
- *Arm 2*: No induction (i.e., 100 ml of normal saline) will be administered intravenously, at 0 and 4 days post engraftment.

### Labeling, handling, and storage

Packaging and labeling of basiliximab and placebo will be performed under responsibility of study pharmacist in accordance with GMP/GCP and national regulatory requirements. All study products will be stored, inventoried, reconciled, and destroyed according to applicable regulations. The product will be labeled according to SFDA regulations as follows:

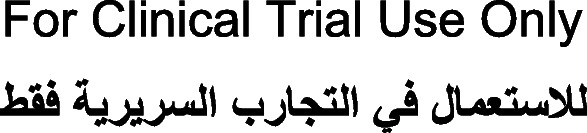



The specific order sentence with (i.e., BASILIXIMAB RAC#2191 177) will be used to the purpose of prescribing the drug for those who have consented to participate in the study.

Medications are properly and safely stored as per King Faisal Specialist Hospital & Research Centre regulations and policies. Medications are stored under necessary conditions: vials refrigerated at 2 to 8 °C (36 to 46 °F).

### Medication dosage and administration

A protocol was developed for immunosuppressive for renal transplant recipients. This protocol represents the overall knowledge, experience, and wisdom of the renal transplant team members according to the best available evidence but is not meant to substitute the general clinical judgment of transplant practitioners. All transplant team members are expected to follow this protocol and deviation from it is only allowed when clinically justified and after consultation with other team members. The 2016 September revision will be followed (Additional file 1).

The placebo is the solution for injection [i.e., 100 ml of normal saline] without basiliximab that is going to be prepared by the research team pharmacist.

### Concomitant medications

Primary immunosuppressive therapy with tacrolimus, anti-proliferative mycophenolate or azathioprine, and steroids. Those medications shall be started 3 days prior to the scheduled day of transplant. Other agents are prohibited.

## Study end points

A. Primary outcomes
  - Biopsy-proven acute rejection within first year after transplant
B. Secondary outcomes
  1. One-year patient and graft survival
  2. Emergence of de novo donor-specific antibodies (DSAs)
  3. Composite of measured glomerular filtration rate (GFR) < 60 mL/min/1.73 m^2^ at month 6 and/or 12
  4. A decrease in measured GFR ≥ 10 mL/min/1.73 m^2^ from month 3 to month 12
  5. Time to first acute rejection
  6. Type and severity of rejection, requirement of antilymphocyte antibody therapy, or steroid-resistant rejection
  7. Clinically treated acute rejection episodes
  8. Number of acute rejection episodes
  9. Any infection (CMV, BKV, urinary tract infection, etc.)
  10. Patient and graft survival beyond 1 year
  11. To assess the difference in calculated and measured GFR
  12. Calculated glomerular filtration rate by Cockcroft-Gault equation
  13. To determine the absolute and relative number of CD25 receptors on T cells at the end of each dosing interval
  14. New onset diabetes after transplant
  15. Slow/delayed graft function (delayed graft function will be defined as the need for dialysis within the first week after transplantation. Slow graft function was defined as a serum creatinine level exceeding 3.0 mg per deciliter on day 5 that did not require treatment with dialysis) [19]

## Assessment of compliance

For maintenance medication following transplant:

1. Tacrolimus (FK) compliance will be summarized by the proportion of subjects who comply between 80 and 120% during each of the following periods (in days): ≤ 28 (≤ 1 month), 29–84 (2–3 months), 85–168 (4–6 months), 169–252 (7–9 months), and 253–364 (9–12 months). Subjects will be instructed to return any unused drug in the original container at post-baseline study visits. Then, returned medication will be reconciled by the study investigator(s) in order to monitor the subject’s compliance with the medication regimen. Compliance with FK therapy will also be assessed by descriptively summarizing FK trough level at day 5; at weeks 2, 4, and 8; and then at every 3-month intervals starting from month 3.

## Efficacy assessments

1. Serum creatinine will be monitored and collected at the following time intervals: at baseline and at weeks 2, 4, 12, 24, 36, and 48. Liver function tests, complete blood count and hematology, chemistry, and other laboratory tests will be collected
2. Biopsy will be carried out based on the discretion of the primary treating consultant (see Appendix). All biopsy will be interpreted according to Banff classification [20] by independent pathologist

## Safety assessment

Safety and tolerability of the study medications including all side effects. Those will include any side effect that occurs upon initiation of therapy until 30 days after completion of therapy. An adverse effect is any untoward medical occurrence in a clinical investigation subject administered a medicinal product and which does not necessarily have a causal relationship with this treatment. An adverse effect can therefore be any unfavorable and unintended sign, symptom, or disease temporally associated with the use of a medicinal product, whether or not considered related to the medicinal product. Active ADR surveillance will be considered.

Adverse events and serious adverse events will be reported to KFSHRC Research Advisory Council and Saudi Food & Drug Authority, respectively, following their standard policies.

## Maintenance protocol for low-risk patients

The maintenance immunosuppression will comprise tacrolimus (Prograf® or envarsus® + mycophenolate mofetil (Cellcept®) + Prednisone):

- *Tacrolimus (FK-506)*
  - ➢ Recipients for induction with basiliximab or no induction receive 0.1 mg/kg/day in two divided doses orally starting 2–4 doses prior to transplantation
  - ➢ Three divided doses (0600, 1400, 2200 h) are given to patients who require ≥15 mg/day to reach a therapeutic trough level.
- *Mycophenolate Mofetil (MMF)*
  - Target dose: 1.5 g/day in two divided doses po:
  - ➢ All recipients receive at least two doses prior to transplant (the day before at 2100 h and the morning of transplant at 0600 h)
  - ➢ In case of side effects (leukopenia, thrombocytopenia), consider other potential culprits in addition to MMF (Valganciclovir, Ganciclovir, Septra®). Adjust or decrease doses accordingly (adjust mycophenolate mofetil dose first and if no response, proceed to adjustment of the doses of Septra® and/or Valcyte®). In case of gastrointestinal side effects (diarrhea, abdominal discomfort) related to mycophenolate mofetil, consider administering the dose with food, decreasing the dose or splitting the total dose into three divided doses.

### Corticosteroids (Solumedrol IV, prednisone po)

Adults: (BW > 25 kg) recipients receive corticosteroids according to the following schedule:

### Corticosteroids’ maintenance protocol

Applicable to patients without early rejection episodes (within the first 3 months post engraftment)

**Table:** 

**Day/week/month**	**Dose**
Day 0	Methylprednisolone 250 mg IV
Day 1	Methylprednisolone 80 mg IV
Day 2	Prednisone 60 mg po
Day 3	Prednisone 50 mg po
Day 4	Prednisone 40 mg po
Day 5	Prednisone 30 mg po
Day 6 to day 14	Prednisone 20 mg po daily
3rd week	Prednisone 17.5 mg po daily
4th week	Prednisone 15 mg po daily
5th week	Prednisone 12.5 mg po daily
6th week	Prednisone 10 mg po daily
7th week	Prednisone 7.5 mg po daily
8th week and on	Prednisone 5 mg po daily
4th to 6th months	Prednisone may be tapered down to 2.5 mg every day or every other day

### Immediate-release tacrolimus (Prograf®) to LCPT long-acting tacrolimus (Envarsus®) conversion

Conversion from Prograf® to Envarsus® may be considered only when all of the following criteria are fulfilled:

1. Eighteen- to 35-year-old recipients with history of biopsy-proven rejection due to non-adherence to immunosuppressants
2. Stable graft function
  Dosing conversion from Prograf® (immediate-release) to once daily Envarsus® should be on a 1:0.7 (mg:mg) total daily dose basis and the Envarsus® maintenance dose should, therefore, be 25–30% less than the Prograf® dose, e.g., a patient who is receiving 1 mg BID tacrolimus (immediate release) should be converted to 1.5 mg tacrolimus LCPT (Envarsus) once daily.

Monitor 24-h trough similarly to the traditional 12-h trough.

### Therapeutic drug monitoring

#### Therapeutic drug Levels

##### Tacrolimus levels

The following whole blood 12-h (or 24-h in case of LCPT tacrolimus) trough levels should be targeted in relation to timing after transplant:

- ➢ 0–30 days:
  - ◦ Target whole blood 12-h trough levels: 8–10 ng/ml
- ➢ > 30 days:
  - ◦ Target whole blood 12-h trough levels: 6–8 ng/ml
    - For newly transplanted patients, tacrolimus trough level will be ordered after the 4th dose.
    - Whole blood 24-h tacrolimus levels should be used for patients on once daily long acting tacrolimus following the same targets

### Antiviral prophylaxis

#### CMV prophylaxis (universal prophylaxis)

- CMV-negative kidney transplant recipients of CMV-positive kidneys receive oral valganciclovir 450 mg/day (adjusted to renal function), for 6 months‡ [21, 22].
- CMV-negative kidney transplant recipients of CMV-negative kidneys receive no prophylaxis [22].
- All CMV-positive renal transplant recipients receive valganciclovir 900–450 mg orally once daily (adjusted to renal function), for 3 months [22].

#### EBV prophylaxis

- EBV-negative kidney transplant recipients of EBV-positive kidneys receive valganciclovir 450 mg orally once daily (adjusted to renal function), for 6 months.
- *Valganciclovir dose is adjusted according to renal function as follows:*

**Table:** 

Cr Cl (ml/min/)*	**Dose**
> 60	No adjustment
40–59	450 mg daily
25–39	450 mg q 48 h
10–24	450 mg twice/week
< 10	Not recommended, use gancilcovir IV 1.25 mg/kg

*Using CG equation and for patients aged > 16 years old

### Anti-bacterial prophylaxis

#### Trimethoprim-sulfamethoxazole (Septra®, Bactrim®)

- All recipients will receive one single strength trimethoprim-sulfamethoxazole tablet po every day (adjusted to renal function) for 6–9 months based on the burden of immunosuppression [23].
- Patients with recurrent urinary tract infections may continue Septra® or other antibiotic prophylaxis for the duration of the graft survival.
- Patients who are allergic to Sulfa or who have documented (glucose 6 phosphate dehydrogenase (G6PD) deficiency will receive pentamidine inhalation 300 mg every month or dapsone 100 mg daily for 6 months [23]. UTI prophylaxis (oral ciprofloxacin or cefuroxime) should be continued in these patients for 6 months at least [24].
- Dose of trimethoprim-sulfamethoxazole will be adjusted to single dose three times per week in patients with GFR ≤ 30 ml/min

### Surgical prophylaxis

- All recipients of living donor organs will receive single dose of IV cefazolin 1 g at the time of induction [25–27].

### Tuberculosis prophylaxis

- All recipients who are PPD skin test positive or donor PPD-negative recipients of kidneys from PPD-positive donors will receive isoniazide (INH) 300 mg po along with vitamin B6 25 mg po every day for 9 months [22, 28].

### Antifungal prophylaxis

- This is aimed predominantly at mucocutaneous Candida infection during multiple high dose steroid therapy and/or prolonged courses of antimicrobial therapy. Patients at risk of mucocutaneous Candida infection receive Nystatin (Mycostatin®) 500,000 IU swish and swallow 3–4 times per day for adults or clotrimazole torches for the duration of high dose steroid or antimicrobial therapy (i.e., ≥ 20 mg prednisone or equivalent) [29, 30].

## Data collection and management

Eligible patients will be identified through electronic pharmacy system and via scheduled transplant/admission date, or from the kidney transplant clinic. Clinical data will be collected using standardized electronic case report form (eCRF) (Additional file 2), through direct patient interview, Integrated Clinical Information System (ICIS), or electronic Medication Administration Records (eMAR) and chart review.

For each enrolled subject, the eCRF will be completed and electronically signed by the principal investigator. Study data will be collected and managed using REDCap® electronic data capture tools hosted at KFSHRC [31]. In case of drop-out, it should be clearly documented. The research personnel will ensure the completeness and accuracy of the data reported in the eCRF. All information will be saved accordance with International Conference on Harmonisation of Good Clinical Practice (ICH-GCP) which is an international ethical and scientific quality standard and each applicable regulatory agency. Study documents and data will be saved in specific locked password protected file and/or personal computer.

### Informed consent

Investigators will ensure that subjects or their legally acceptable representatives are clearly and fully informed about the purpose, potential risks, and other critical issues regarding clinical trials in which they volunteer to participate. The investigator(s) will provide the subject or legally acceptable representative with a copy of the consent form and written information about the study. The investigator(s) should allow time necessary for the subject or subject’s legally acceptable representative to inquire about the details of the study. Subjects will be told that they are free to not to take part in the study and may withdraw their consent at any time for any reason. The informed consent must be signed and personally dated by the subject or the subject’s legally acceptable representative, by the person who conducted the informed consent procedure, and a witness. A copy of the signed ICF will be provided to the subject, a copy will be kept in the patient file, and one will be kept with the principal investigator.

### Ethical consideration and patient confidentiality

Written informed consent will be obtained from all subjects prior to enrolment. This study will be conducted in accordance with the latest version of the Declaration of Helsinki and Good Clinical Practice [32] (as amended in Edinburgh, Tokyo, Venice, Hong Kong, and South Africa) [33], the policies and procedures of Research Advisory Council (RAC) of the KFSHRC, Saudi Food & Drug Authority, and the laws of Saudi Arabia. The investigators assure that subjects’ anonymity will be maintained and that their identities are protected from unauthorized parties. Subject names will not be supplied to parties other than the study investigators. Study data and findings will be stored on a computer and will be handled in strictest confidence in accordance with local data protection laws. Study records will be maintained by the principal investigator in secure location.

## Glossary of abbreviations and definition of terms (Tables 1 and 2)

**Table 1:** Abbreviations

Term	Definition
**ALT**	Alanine aminotransferase
**AE**	Adverse event
**ANC**	Absolute neutrophil count
**ANOVA**	Analysis of variance
**AST**	Aspartate aminotransferase
**AZA**	Azathioprine
**BCAR**	Biopsy-proven acute rejection
**BMI**	Body mass index
**BP**	Blood pressure
**CI**	Confidence interval
**CMV**	Cytomegalovirus
**CNI**	Calcineurin inhibitor
**CSA**	Cyclosporine
**EBV**	Epstein-Barr virus
**eGFR**	Estimated glomerular filtration rate
**FK**	Tacrolimus
**HBV**	Hepatitis B virus
**HIV**	Human immunodeficiency virus
**IVIG**	Intravenous immune globulin
**MMF**	Mycophenolate
**PK**	Pharmacokinetic(S)
**PRA**	Panel reactive antibodies
**PTDM**	Post-transplant diabetes mellitus
**PTLD**	Post-transplant lymphoproliferative disorder
**SrCr**	Serum creatinine

**Table 2:** Definitions

Patient variable	Definition		
**Adverse events**	An AE is any untoward medical occurrence in a clinical investigation subject administered a medicinal product and which does not necessarily have a causal relationship with this treatment. An AE can therefore be any unfavorable and unintended sign, symptom, or disease temporally associated with the use of a medicinal product, whether or not considered related to the medicinal product. AEs also include the following: Pre- or post-treatment complications that occur as a result of protocol mandated procedure (e.g., such as venipuncture, biopsy) during or after screening (before the administration of study investigational medicinal product). Any pre-existing condition that increases in severity, or changes in nature during or as a consequence of the study investigational medicinal product phase of a human clinical trial, will also be considered an AE.		
**Acute rejection**	*Acute rejection* will be defined as a clinico-pathological event requiring clinical evidence and biopsy confirmation. Allograft biopsies will be evaluated for the presence and severity of acute rejection by a blinded central independent pathologist using Banff 97 working classification of kidney transplant pathology. In the analyses of acute rejection, the biopsy interpretation and grading by the central pathologist will supersede local interpretation. Biopsies performed for suspected acute rejection that do not fully meet the criteria but are interpreted by the central pathologist as acute rejection and result in treatment for acute rejection, will be counted as acute rejection. *Subclinical rejection* is defined as histological findings by the central pathologist consistent with acute rejection, but lacking its clinical correlate. *Steroid-resistant acute rejection* is defined as the use of lymphocyte-depletion therapy following treatment with corticosteroids.		
**Bone profile**	Serum calcium, albumin, phosphorus, and magnesium		
**Chemistry**	Glucose, potassium, sodium, chloride		
**Chronic allograft nephropathy (CAN)**	Biopsy-proven CAN will be determined by a central histopathologist using the Banff working classification of kidney transplant pathology.		
**Coagulation**	INR, prothrombin time (PT), activated partial thromboplastin time (APTT)		
**Creatinine clearance**	Creatinine clearance is calculated by the Cockcroft-Gault equation using ideal body weight (IBW). Male: CLcr (mL/min) = [140 − age (years)] × BW (kg)/72 × Scr Female: CLcr (mL/min) = [140 − age (years)] × BW (kg) × 0.85/72 × Scr		
**Criteria for performing an allograft biopsy to assess for acute rejection**	1. An unexplained rise of serum creatinine ≥25% from baseline creatinine 2. An unexplained decreased urine output 3. A serum creatinine that remains elevated within 14 days post-transplantation and clinical suspicion of acute rejection exists		
**delayed graft function (DGF)**	A subject will be determined to have DGF if the subject is treated with dialysis within the first week (days 1–7) post transplantation.		
**Graft Loss**	Graft loss is defined as either functional loss or physical loss. Functional loss will be defined as a sustained level of SCr ≥ 6.0 mg/dL (530 μmol/L) as determined by the central laboratory for ≥4 weeks or ≥ 56 consecutive days of dialysis, or impairment of renal function to such a degree that the subject undergoes re-transplant		
**Hematology**	Hematocrit, hemoglobin (Hb), platelet count, red blood cell count (RBC), white blood cell count (WBC) with differential (absolute and percentage) including lymphocytes, monocytes, neutrophils, eosinophils, basophils, reticulocyte count, and mean corpuscular volume (MCV).		
**Hypertension**	Hypertension will be as defined in this study according to the Eighth Report of the Joint National Committee on the Prevention, Detection, Evaluation, and Treatment of High Blood Pressure [34, 35] for subjects with chronic kidney disease. This definition is based upon systolic blood pressure (SBP) ≥ 130 mmHg or diastolic blood pressure (DBP) ≥ 80 mmHg. In addition, all subjects who have a SBP < 130 mmHg and a DBP < 80 mmHg who are receiving an antihypertensive medication(s) for the indication of hypertension or with a medical history of hypertension are included in this definition.		
**Immediate graft function (IGF)**	A subject will be determined to have IGF if SCr at Day 5 < 3.0 mg/dL (i.e., 260 μmol/L) and if the subject did not have DGF.		
**Liver enzymes**	Alanine aminotransferase (ALT/SGPT), aspartate aminotransferase (AST/SGOT), albumin, alkaline phosphatase, direct bilirubin (screening only), total bilirubin, lipase		
**Measures of acute rejection**	Acute rejection will be defined as a clinico-pathological event requiring clinical evidence and biopsy confirmation. Allograft biopsies will be evaluated for the presence and severity of acute rejection by a central independent pathologist using the most recent Banff working classification of kidney transplant pathology.		
**Post-transplant diabetes mellitus (PTDM)**	PTDM will be defined according to the definition set forth by a recent international consensus guideline [36–40]. These criteria are summarized as: • Symptoms of diabetes plus casual plasma glucose (PG) concentration ≥ 200 mg/dL (11.1 mmol/L) OR • fasting plasma glucose (FPG) ≥ 126 mg/dL (7.0 mmol/L) OR • 2-h Plasma Glucose ≥ 200 mg/dL (11.1 mmol/L) during an oral glucose tolerance test (GTT) AND • A confirmatory laboratory test based on measurements of venous PG must be done on another day in the absence of unequivocal hyperglycemia accompanied by acute metabolic decompensation.		
**Stages of chronic kidney disease**	The stages of chronic kidney disease are defined as set forth in the NKF-K/DOQI guidelines.		
	**Stage**	**Description**	**GFR (mL/min/1.73 m**^**2**^**)**
	**1**	Kidney damage with normal or ↑ eGFR	≥ 90
	**2**	Kidney damage with mild ↓ GFR	60–89
	**3**	Moderate ↓ GFR	30–59
	**4**	Severe ↓ GFR	15–29
	**5**	Kidney failure	< 15 (or dialysis)
**Slow graft function (SGF)**	A subject will be determined to have SGF if SCr at day 5 ≥ 3.0 mg/dL (i.e., 260 μmol/L) and if the subject did not have DGF.		

### Supplementary Information

**Additional file 1.** Protocol for Immunosuppressive for Renal Transplant Recipients.

**Additional file 2.** Informed consent.

## Data Availability

Not applicable.

## Appendix

**Table 3:** Schedule of Assessment

Schedule of Assessment								
Procedure	Screening visit	Baseline	Week 2	Week 4	Month 3 (week 12)	Month 6 (week 24)	Month 9 (week 36)	Month 12 (week 48)
**Informed consent**	x							
**Medical history and demographic data**	x							
**Review inclusion & exclusion criteria**	x							
**Donor data collection**	x							
**Laboratory**	x	x	x	x	x	x	x	x
**Serology**	x							
**Donor-specific antibodies (DSAs)**	x				x	x	x	x
**Biopsy**			x					
**2nd biopsy and treatment of rejection**	**When clinically indicated at the discretion of treating physician**							
