# Supplementary material for: Standard induction with basiliximab versus no induction in low immunological risk kidney transplant recipients: study protocol for a randomized controlled trial
Source: Trials. 2021 Jun 24;22:414. doi: 10.1186/s13063-021-05253-1 (PMC8223264; doi:10.1186/s13063-021-05253-1)
Supplement: Supplementary file 1 — Additional file 1. Protocol for Immunosuppressive for Renal Transplant Recipients. [file 13063_2021_5253_MOESM1_ESM.docx]

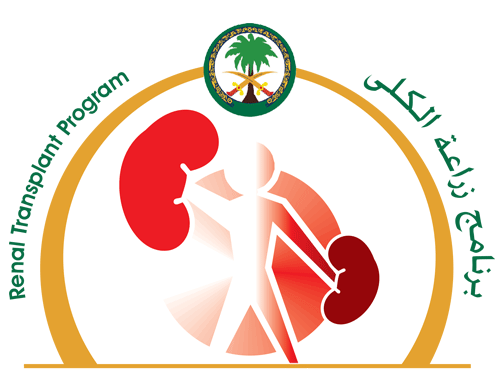


Immunosuppressive

and

Other Supplementary Protocols for

Renal Transplant Recipients

## *September 2016*

**On behalf of the Kidney and Pancreas Transplantation Team Members at**

**King Faisal Specialist Hospital and Research Centre**

**Members:**

Ibrahim Al-Ahmadi, MD, Consultant Surgeon, KPTS , Chair, Department of Kidney and Pancreas Transplantation (KPT)

Ammar Abdulbaki, MD, ABIM, Consultant Transplant Nephrologist, Section Head, Adult Transplant Nephrology, Department of Kidney and Pancreas Transplantation (KPT)

Khaled Almeshari, MD, ABIM, FAST, Consultant Transplant Nephrologist. Adult Transplant Nephrology, Department of Kidney and Pancreas Transplantation (KPT)

Jens, Brockman, MD, Consultant Transplant surgeon, Head Section, Kidney and Pancreas Transplant Surgery, KPT

Ahmed Al-Jedai, PharmD, Director, Consultant Clinical Pharmacists, Pharmaceutical Care Division

Hassan Al Eid, MD, Consultant Transplant Nephrologist, KPT

Tariq Ali, MD, Consultant Transplant Nephrologist, KPT

Hazem El Gamal, MD, Associate Consultant Transplant Nephrologist, KPT

Yaser Shah, MD, Consultant Transplant Nephrologist, KPT

Syed Raza, MD, Consultant Transplant Surgeon, KPTS, KPT

Amira Alabbasi, MD, Consultant Transplant Surgeon, KPTS, KPT

Hani Alahdal, MD, Associate Consultant Transplant Surgeon, KPTS, KPT

Ehab Hassan Hammad, MD, Assistant Consultant Transplant Nephrologist, KPT

Ihab Ibrahim, MD, Assistant Consultant Transplant Nephrologist, KPT

Mohamad Elhaj Hussein, MD, Assistant Consultant Transplant Nephrologist, KPT

Abdulrahman Alkhan, MD, Assistant Consultant Transplant Nephrologist, KPT

Shahid Khan, MD, Assistant Consultant Surgeon, KPTS, KPT

Asad Bashir, MD, Assistant Consultant Surgeon, KPTS, KPT

Delal Alkortas, Pharm.D., Pharmacy Services

Dema Alissa, B.Sc. Pharm., SSC_PhP, MBA, Pharmacy Services

Zinah Alabdulkarim, B.Sc. Pharm., Resident, Pharmacy Services

| **Table of contents** | | **Page** |
| --- | --- | --- |
|  | Introduction | 4 |
| Chapter I | Pediatric Immunosuppressive Protocol | 6 |
| Chapter II | Adult Immunosuppressive Protocol | 11 |
| Chapter III | Antimicrobial Prophylaxis | 21 |
| Chapter IV | Supplementary Medications | 29 |
| Chapter V | Infertility and Pregnancy | 31 |
| Chapter VI | Minimization and Protocol Biopsies | 35 |
| Chapter VII | Monitoring and Management of De Novo HLA DSA | 38 |
| Chapter VIII | Monitoring and Management of BKV nephropathy | 40 |
| Chapter IX | Management of HLA and ABO incompatible transplantation | 42 |
| Chapter X | Adult vaccination schedule | 45 |
| Chapter XI | Score point system for Deceased Donor Kidney Allocation | 47 |
|  | Thymoglobulin Guidelines  Alemtuzumab Guidelines | 49  51 |
|  | Intravenous Immune Globulin (IVIG) guidelines | 52 |
|  |  |  |
|  |  |  |

**Disclaimer:**

**This protocol represents the overall knowledge, experience and wisdom of the renal transplant team members according to the best available evidence but is not meant to substitute the general clinical judgment of transplant practitioners. All transplant team members are expected to follow this protocol and deviation from it is only allowed when clinically justified and after consultation with other team members.**

**Introduction:**

The immunosuppressive protocol for renal transplant recipients consists of Induction and Maintenance Protocols.

**Induction Protocol** is designed for a select group of renal transplant recipients who are at risk for immunologic injury.

It is defined as the use of polyclonal (T-lymphocyte-depleting: Thymoglobulin®) or monoclonal (non-lymphocyte-depleting: Basiliximab®) antibody preparations in the pre and the early post-transplant periods of kidney transplantation.

**Maintenance Protocol** is Tacrolimus-based regimen with adjuvant agents.

**CHAPTER I**

**PEDIATRIC IMMUNOSUPPRESSIVE PROTOCOL**

**Chapter I. Pediatric Immunosuppressive Protocol**

**Definition:**

Pediatric renal transplant recipients are defined as < 14 years of age at the time of transplantation.

**Induction:**

**LD transplants**

LD recipients of zero HLA mismatched grafts receive no induction therapy.

All LD recipients otherwise receive induction with Basiliximab except the following:

- HLA incompatible
- ABO incompatible
- Delayed graft function

They receive induction with Thymoglobulin® *(Please refer to Thymoglobulin® Guidelines)*

**DD transplants**

All DD recipients receive induction with Thymoglobulin® *(Please refer to Thymoglobulin® Guidelines).*

**Maintenance Therapy:**

All recipients receive maintenance immunosuppressive regimen consisting of Tacrolimus, MMF, and prednisone.

- ***Basiliximab (Simulect^®^)***
  - **Pediatrics:** 10 mg IVPB for weight < 35 kg, and 20 mg for weight ≥ 35 kg, one dose immediately prior to reperfusion and the second dose is administered on the 4^th^ post-operative day.
- ***Corticosteroids (Methylprednisolone IV, prednisone PO)***
  - **Pediatrics: (BW ≤25 kg)** LD & DD recipients receive Corticosteroids according to the following schedule:

| **Day** | **mg/kg/Day** |
| --- | --- |
| **0** | **Methylprednisolone 10 IV (maximum dose of 250 mg)** |
| **1-2** | **Prednisone 2.0 (oral)** |
| **3-6** | **Prednisone 1.5** |
| **7-8** | **Prednisone 1.0** |
| **9-15** | **Prednisone 0.75** |
| **16-30** | **Prednisone 0.5** |
| **31-45** | **Prednisone 0.4** |
| **46-60** | **Prednisone 0.3** |
| **61-90** | **Prednisone 0.2** |
| **91-120** | **Prednisone 0.1** |

| **Day** | **mg/kg/Every Other Day** |
| --- | --- |
| **> 120** | **Prednisone 0.1** |

Minimum dose: 2.5 mg/day for children ≤25 kg at any stage of the taper.

- - **Tacrolimus:**

0.15 – 0.3 mg/kg/day in two divided doses or three divided doses po as follows :

- - - LD recipients receive 0.15 mg/kg/day, 48 hours prior to transplant surgery.
    - Recipients of DD kidney transplants receive 0.15 mg/kg/day, once immediate graft function is established.
    - Recipients who do not reach therapeutic blood levels on maximum doses of twice a day (BID) regimen by day 5 are switched to three times a day (TID) regimen.
    - Recipients of body weight < 15 kg receive thrice/day regimen.

**Therapeutic Drug Monitoring and dose calculation:**

- - **Tacrolimus levels**:

Target whole blood 12-hour trough level using (LC/MS/MS method):

8 - 10 ng/ml 0 – 30 days

6 – 8 ng/ml 31 – 90 days

4 – 6 ng/ml > 90 days

- - **MMF:**

1. 600 mg/m^2^/dose BID or 400mg/m^2^/dose TID to a maximum of 2 gm/day.
2. Patients may be given BID or TID regimen
   - - LD recipients receive two doses prior to transplant (the day before at 2100 hours and the morning of transplant at 0600 hours)
     - DD recipients receive one dose prior to transplant surgery

**CMV Prophylaxis:**

**Pediatric patients dose of Valganciclovir is calculated as follows:**

Once daily dose (mg) = 7 x body surface area x Cl_cr_

Cl_cr_ (modified Schwartz: mL/min/1.73 m^2^) = [k x height (cm)] ÷ SCr (mg/dL) where K =

- 0.45 in patients <2 years
- 0.55 in boys age 2 to <13 years
- 0.55 in girls age 2 – 16 years
- 0.7 in boys age 13 – 16 years

If the calculated Cl_cr_ is >150 mL/minute/1.73 m^2^, use 150 to calculate the dose. Round to nearest 25; maximum 900 mg/day.

**PJP Prophylaxis:**

Patients who are not G6PD deficient and have no allergy to sulfa drugs should receive Septra^®^ 80 mg/m^2^ once daily for 12 months

Alternative for G6PD and sulfa allergy

**CHAPTER II**

**ADULT IMMUNOSUPPRESSIVE PROTOCOL**

**CHAPTER II – Adult Immunosuppressive Protocol**

**Definition:**

Adult renal transplant recipients are defined as ≥ 14 years of age at the time of transplantation.

**Induction Protocol:**

The choice of the induction agent (polyclonal or monoclonal) is based on the stratification of immunologic risks (high or low risk) as follows:

### I. High Immunologic Risks LD & DD (Induction with Thymoglobulin®)

1. HLA incompatible* and/or ABO incompatible patients
2. Sensitized patients (positive DSA and/or positive non-DSA HLA antibodies with MFI > 2000)
3. Re-transplants Excluding Zero HLA mismatched
4. Historically positive, currently negative crossmatch Excluding Zero HLA mismatched
5. DD transplants Excluding Zero HLA mismatched
6. Patients with Delayed Graft Function (*DGF)
7. Young adults (14 to 25 years of age) Excluding Zero HLA mismatched
8. > 4 Antigen mismatch (HLA A, B, DR, DQ matching scheme)*
9. Transplant from Child to mother, husband to wife (if previous conception from donor)

**DGF is defined as dialysis-dependent renal allograft dysfunction during the first week of engraftment.*

** HLA incompatibility is defined as positive flow and/or CDC lymphocyte cross match*

* *EBV naïve patients should have anti-viral prophylaxis and monitoring for EBV reactivation. Alternatively, and as per the discretion of the treating physician, induction with Basiliximab might be considered.*

* *All recipients and donors will be routinely typed for HLA A, B, DR, DQ loci. HLA matching scheme will therefore be 8 antigen match/mismatch*

#### Induction Protocol for High Risk Patients

### *Rabbit anti-thymocyte globulin Thymoglobulin^®^:* (Lymphocyte depleting, polyclonal preparation.)

1.5 mg/kg/day, IV infusion through a central line, AV fistula/graft, or peripheral line (*Please refer to Thymoglobulin^®^ Guidelines*). The infusion of the first dose to start prior to reperfusion of the renal allograft. Patients receive three to five doses (total maximum dose of 7.5 mg/kg), the first three of which are given regardless of FK-506 level, T-lymphocyte absolute counts or CD markers. However, subsequent doses will be administered if FK-506 level is still sub-therapeutic and T-lymphocyte counts and/or T-cell markers reflect inadequate T-lymphocyte depletion. the dose can be adjusted for side effects as per Thymoglobulin Guidelines.

In case of DGF following LD transplant, Thymoglobulin^®^ is given after the event occurs, the second dose of Basiliximab will be discontinued and Tacrolimus will be withheld. Tacrolimus is re-introduced once graft function is established (serum creatinine is ≤ 300 μmol or polyuria with a remarkable decline in serum creatinine: ≥ 25% drop from baseline value).

HLA/ABO compatible patients with detected DSA reaching MFI ≥ 1000 will also receive IVIG 1 gram/kg immediately pre-operatively.

**Maintenance Protocol for High Risk Patients**

Tacrolimus + MMF + prednisone.

- ***Tacrolimus (FK-506)***

Recipients for induction with Lymphocyte depleting preparation (i.e. Thymoglobulin) receive 0.1 – 0.2 mg/kg/day in two divided doses orally starting post-operatively once graft function is established.

Three divided doses (0600, 1400, 2200 hours) are given to patients who require ≥ 15 mg/day to reach a therapeutic trough level.

- ***Mycophenolate Mofetil (MMF)***

1.5 –gm/day PO in two divided doses:

- LD recipients receive two doses prior to transplant (the day before at 2100 hours and the morning of transplant at 0600 hours)
- DD recipients receive one dose prior to transplant surgery
- In case of side-effects (leukopenia, thrombocytopenia), consider other potential culprits in addition to MMF (Valganciclovir, Ganciclovir, Septra). Adjust or decrease doses accordingly (adjust MMF dose first and if no response, proceed to adjustment of the doses of Septra and/or Valcyte). In case of GI side effects (diarrhea, abdominal discomfort) related to MMF, consider administering the dose with food, decreasing the dose or splitting the total dose into three divided doses.
- High risk patients or those with prior rejection or those on cyclosporine can receive 2gm/day in 2 divided doses

**II. Low Immunologic Risk LD candidates (induction with Basiliximab)**

Adult renal transplant recipients who fulfill the following characteristics:

1. First (primary) transplant
2. ≤ 4 Antigen mismatch (HLA A, B, DR, DQ matching scheme);
3. Negative HLA Ab screening

**Induction Protocol for Low-Risk Patients:**

***Basiliximab (Simulect^®^):*** (Non-lymphocyte depleting monoclonal antibody that targets IL2 receptors on activated T-lymphocyte).

Two doses, 20 mg IVPB, at 0 and 4 days post engraftment. The first dose to be given prior to reperfusion.

**Induction protocol for Recipients of HLA identical or zero mismatched transplants:**

LD recipients of HLA identical or zero mismatched allografts receive no induction therapy. However, if they are sensitized/ABO compatible, they receive induction with Basiliximab and if they are recipients of ABO incompatible transplants, they receive induction with Thymoglobulin^®^.

**Maintenance Protocol for Low-Risk Patients:**

Tacrolimus + MMF + Prednisone

- ***Tacrolimus (FK-506)***
- Recipients for induction with Basiliximab or no induction receive 0.1 – 0.2 mg/kg/day in two divided doses orally starting 2 – 4 doses prior to transplantation
- Three divided doses (0600, 1400, 2200 hours) are given to patients who require ≥ 15 mg/day to reach a therapeutic trough level.
- ***Mycophenolate Mofetil (MMF)***

1.5 – 2.0 gm/day in two divided doses po:

- LD recipients receive two doses prior to transplant (the day before at 2100 hours and the morning of transplant at 0600 hours)
- DD recipients receive one dose prior to transplant surgery
- In case of side-effects (leukopenia, thrombocytopenia), consider other potential culprits in addition to MMF (Valganciclovir, Ganciclovir, Septra). Adjust or decrease doses accordingly (adjust MMF dose first and if no response, proceed to adjustment of the doses of Septra and/or Valcyte). In case of GI side effects (diarrhea, abdominal discomfort) related to MMF, consider administering the dose with food, decreasing the dose or splitting the total dose into three divided doses.
- ***Corticosteroids (Solumedrol IV, prednisone po)***

**Adults: (BW > 25 kg)** LD & DD recipients receive Corticosteroids according to the following schedule:

**Corticosteriods Maintenance Protocol**

Applicable to patients without early rejection episodes (within the first 3 months post engraftment)

| **Day / Week / Month** | **Dose** |
| --- | --- |
| Day 0 | Methylprednisolone 250 mg IV |
| Day 1 | Methylprednisolone 125 mg IV |
| Day 2 | Prednisone 60 mg po |
| Day 3 | Prednisone 50 mg po |
| Day 4 | Prednisone 40 mg po |
| Day 5 | Prednisone 30 mg po |
| Day 6 and on | Prednisone 20 mg po daily |
| 2^nd^ week | Prednisone 17.5 mg po daily |
| 3^rd^ week | Prednisone 15 mg po daily |
| 4^th^ week | Prednisone 12.5 mg po daily |
| 5^th^ week | Prednisone 10 mg po daily |
| 6^th^ week | Prednisone 7.5 mg po daily |
| 7^th^ week and on | Prednisone 5 mg po daily |
| 4^th^ – 6^th^ months | Prednisone may be tapered down to 2.5 mg every day or every other day |

Different regimen of steroid taper or withdrawal may be considered on individualized basis when indicated

### Conversion to Other Immunosuppresants

**Tacrolimus to Sirolimus**

Conversion from tacrolimus to Sirolimus-based immunosuppression may be considered on individualized basis when indicated

The main indications for conversions are:

1. A biopsy proven significant interstitial fibrosis (IF), tubular atrophy (TA), fibro-intimal hyperplasia (FIH), and CNI toxicity

**Pre-requisites for conversion are:**

- **Proteinuria < 1 gm in a 24 hour urine collection**
- **Serum creatinine ≤ 250 μmol/L (GFR ≥30 – 40 cc/m)**

1. Kaposi sarcoma
2. As part of strategy for lowering immunosuppression in the context of serious viral infection or malignancy

### Conversion should generally be avoided in:

- Patients with previous episode(s) of rejection
- HLA or ABO incompatible transplants

**Immediate-release tacrolimus (Prograf®) to LCPT long-acting tacrolimus (Envarsus®) conversion**

Conversion from Prograf® to Envarsus® may be considered only when all of the following criteria are fulfilled:

1. 18-35 years old recipients with history of biopsy proven rejection due to non-adherence to immunosuppressants
2. Stable graft function

- Dosing conversion from Prograf® (immediate-release) to once daily Envarsus® should be on a 1:0.7 (mg:mg) total daily dose basis and the Envarsus® maintenance dose should, therefore, be 25-30% less than the Prograf® dose. e.g. a patient who is receiving 1 mg BID tacrolimus (immediate release) should be converted to 1.5 mg tacrolimus LCPT (Envarsus) once daily.

Monitor 24-hr trough similarly to the traditional 12-hr trough.

### Conversion should generally be avoided in:

1. Donation after cardiac death
2. Ongoing acute cellular or antibody mediated rejection
3. Patients with a BMI <18 kg/m2 or >40 kg/m2
4. Patients who received or expected to receive sirolimus, everolimus, azathioprine or cyclophosphamide
5. Patients with eGFR <30 mL/min
6. Pediatric patients

**Therapeutic Drug Monitoring**

- ***Therapeutic Drug Levels:***

**Tacrolimus levels:**

The following **whole blood 12-hour (or 24-hr in case of LCPT tacrolimus) trough levels should be targeted in**

**relation to timing after transplant:**

- 0-3 months:
  - Target whole blood 12-hour trough levels:

6-10 ng/ml

- > 3 months:
  - Target whole blood 12-hour trough levels:

5-8 ng/ml

- **For newly transplanted patients, Tacrolimus trough level will be ordered after the 4^th^ dose.**
- **Maintain high risk patients (ABO incompatible and highly sensitized patients on 6-10 ng/ml beyond the 3 months period)**
- **Whole blood 24-hour Tacrolimus levels should be used for Patients on once daily long acting Tacrolimus following the same targets**

**Sirolimus levels:**

- Target whole blood 24-hour trough level:

5 – 10 ng/ml

**CHAPTER III**

**ANTIMICROBIAL PROPHYLAXIS**

**Chapter III. Antimicrobial Prophylactic Protocol**

**Antiviral Prophylaxis:**

**CMV Prophylaxis (Universal Prophylaxis):**

- CMV negative kidney transplant recipients of CMV positive kidneys receive oral Valganciclovir 450 mg/day (adjusted to renal function), for **six** **months**‡.
- CMV negative kidney transplant recipients of CMV negative kidneys receive no prophylaxis.
- All CMV positive renal transplant recipients receive Valganciclovir 450 mg orally once daily (adjusted to renal function), for **three months**‡**.**
- CMV negative kidney transplant patients who are ≥ 60 years and receive CMV negative kidneys receive acyclovir 400 mg orally twice daily (adjusted to renal function), for **three months**‡**.**
- All renal transplant recipients requiring Antilymphocyte Antibodies (i.e. Thymoglobulin), receive intravenous Ganciclovir (1.25 – 5 mg/kg/day) adjusted for the first three days while on Thymoglobulin prior to starting oral Valganciclovir .

**EBV Prophylaxis:**

- EBV negative kidney transplant recipients of EBV positive kidneys receive Valganciclovir 450 mg orally once daily (adjusted to renal function), for **six months**‡.
- ‡**Valganciclovir dose is adjusted according to renal function as follows:**

| **Cr Cl (ml/min/)*** | **Dose** |
| --- | --- |
| > 60 | No adjustment |
| 40 - 59 | 450 mg daily |
| 25 - 39 | 450 mg q 48 hours |
| 10 – 24 | 450 mg twice/week |
| < 10 | Not recommended, use gancilcovir IV 1.25 mg/kg |

- * using CG equation and for patients aged > 16 years old

**Anti-bacterial Prophylaxis:**

Trimethoprim-Sulfamethoxazole (Septra®, Bactrim®):

- LD & DD recipients receive one single strength Trimethoprim-sulfamethoxazole tablet po every day (adjusted to renal function) for 9 months.
- Patients with recurrent urinary tract infections may continue Septra or other antibiotic prophylaxis for the duration of the graft survival.
- Patients who are allergic to Sulfa or who have documented G6PD deficiency will receive pentamidine inhalation 300 mg every month or dapsone 100 mg daily for six months. UTI prophylaxis ( oral ciprofloxacin or cefuroxime) should be continued in these patients for 6 months at least.
- Dose of Trimethoprim-Sulfamethoxazole will be adjusted to single dose three times per week in patients with GFR ­< 30 ml/min

**All recipients of deceased donor organs will receive IV Piperacillin/Tazobactam**

**(Tazocin) for the first three days post-transplant starting with day Zero.**

**Tuberculosis Prophylaxis:**

- LD & DD recipients who are PPD skin test positive or LD PPD negative recipients of kidneys from PPD positive donors will receive INH 300 mg po along with Vitamin B6 25 mg po every day for 9 months.
- All DD recipients will receive INH 300 mg po along with Vitamin B6 25 mg po every day for 9 months.

**Antifungal Prophylaxis:**

- This is aimed predominantly at Mucocutaneous Candida infection during multiple high dose steroid therapy and/or prolonged courses of antimicrobial therapy. Patients at risk of Mucocutaneous Candida infection receive Nystatin (Mycostatin^®^) 500,000 IU swish and swallow 3-4 times per day for adults or Clotrimazole trouches for the duration of high dose steroid or antimicrobial therapy.

**Prophylaxis for Thymoglobulin® requiring acute rejection**

1. **Infectious risks associated with Thymoglobulin® as a treatment for acute rejection:**

|  | ***Risk*** |
| --- | --- |
| ***BK virus*** | Increased risk of infection especially with higher doses of ATG administration |
| ***CMV*** | Increased risk of infection |
| ***EBV/PTLD*** | Possible increased risk especially EBV D+/R- although data are mixed |
| ***Fungal infections*** | Increased risk for endemic fungal infections |
| ***HCV/HBV*** | Accelerated HCV replication but no increased rate of hepatic graft injury. Not associated with poor outcomes with HCV+ renal transplant patients.  Possible increased risk of HBV replication in anti-Bc or HBsAg positive patients. |
| ***Pneumocystis*** | Increased risk of infection without appropriate prophylaxis |

1. **Antimicrobial prophylaxis for Thymoglobulin® as a treatment for acute rejection:**

- All recipients who require Thymoglobulin® for treatment of acute rejection will receive the following antimicrobial prophylactic regimen for 3 - 6 months:

1. Valganciclovir 450 mg once po daily adjusted to renal function
2. Septra single strength one po daily adjusted to renal function
3. Lamivudine 100 mg once po daily adjusted to renal function for HBsAg or HBcAb positive recipients, even if viral load is undetectable.

**Prophylaxis for Rituximab® requiring AMR**

Lamivudine 100 mg once po daily adjusted to renal function **OR** Entecavir 0.5 mg once po daily adjusted to renal function (six months) for recipients who are HBsAg positive or HBcAb positive with HBsAb titre of <100, even if viral load is undetectable.

Lamivudine and entecavir doses are adjusted according to renal function as follows:

| Cr Cl (ml/min/1.73m2) | Lamivudine | Entecavir (naïve) | Entecavir (experienced patients) |
| --- | --- | --- | --- |
| 30- 49 ml/ min | 100 mg q 48 hours | 0.5 mg q 48 hours | 1 mg q 48 hours |
| 10- 29 ml/ min | 100 mg q 72 hours | 0.5 mg q 72 hours | 1 mg q 72 hours |
| < 10 ml/ min | 100 mg once weekly after dialysis | 0.5 mg once weekly after dialysis | 1 mg once weekly after dialysis |

Ensure that patient received Pneumococcal vaccination within the 5 years preceding Rituximab administration

**Donors and/or Recipients with HBV infection**

1. **Management of recipients of HBcAb+ donor kidney**
2. **Management of recipients with HBcAb+, HBsAg- and HBsAb- status**

| **Hepatitis B** | |
| --- | --- |
| **Pre-transplant** | Post-transplant |
| **Renal transplant recipient (anti-HBc+ / HBsAg-)** |  |
| Determine anti-HBs status  If negative vaccination course or booster dose  No additional testing or therapy needed pre-transplant | Risk of reactivation is low (0-5%) unless triggered by enhanced IS (e.g. use of Thymoglobulin®). It generally occurs within the first year. Optimal strategy for prevention uncertain  Options include prophylaxis with Lamivudine 100 mg daily x 12 months |

1. **Management of recipients with HBsAg+ status**

| **Hepatitis B** | |
| --- | --- |
| **Pre-transplant** | Post-transplant |
| **Renal transplant candidate/recipient (HBsAg+)** |  |
| Pre-transplant liver biopsy.  Consideration for therapy should be based on published treatment guidelines; Entecavir or Tenofovir is preferred due to high potency, low risk of resistance. | Nucleoside analogue therapy (Entecavir or Tenofovir) indefinitely  In those with low viral load (<2000 IU/mL), Lamivudine 100 mg daily  For live donor transplants, initiate antiviral therapy 1 week prior to transplant; for deceased donor transplants, initiate antiviral therapy immediately when donor identified  Post-transplant monitoring should include liver enzymes, liver function tests and HBV DNA every 3-6 months and abdominal ultrasound every 6-12 months |

**CHAPTER IV**

**SUPPLEMENTARY MEDICATIONS**

**Chapter IV. Supplementary Medications**

**Antihypertensive:**

1. Calcium channel blockers: amlodipine,long acting Nifedipine
2. Beta blockers: atenolol,metoprolol or carvedilol
3. Clonidine
4. * Angiotensin-Converting Enzyme (ACE) inhibitors and Angiotensin-Receptor Blockers (ARB)

**ACE and ARB are avoided during the first 3 months of engraftment except for proteinuria*

**Antihyperlipidemic:**

1. Atorvastatin or simvastatin

**Supplementary Drugs:**

1. Vitamin D
2. Calcium
3. Magnesium oxide
4. Aspirin for high CV risk patients
5. Folic acid

**CHAPTER V**

**INFERTILITY AND PREGNANCY**

**Chapter V. Infertility and Pregnancy**

**Fertility:**

Fertility is commonly restored after successful transplantation.

Adult recipients who are to be started on, or converted to, mTOR-based regimen, and wish to maintain fertility should be counseled regarding the risk of secondary infertility.

mTOR induced infertility is usually reversible with restoration of spermatogenesis and ovulation following discontinuation of the agent

**Contraception:**

Female recipients with child-bearing potential and their partners should be counseled about pregnancy prior to, and as soon as possible after transplantation.

It is strongly recommended that female transplant recipients avoid pregnancy in the first year post transplantation.

The American Society of Transplantation (AST) consensus recommends that pregnancy is allowable if there has been:

- no rejection within the past year
- adequate and stable graft function
- no acute infections that may impact fetal growth and well-being
- maintenance immunosuppression is at stable dosing

Barrier contraception is the method of choice (e.g., condom and/or diaphragm). Oral and injectable hormonal contraceptives are acceptable alternatives. IUD’s are not recommended in transplant recipients.

**Pregnancy:**

Patients are instructed to inform their physician of their intention to become pregnant at least one month prior to the planned conception, to discuss risks and allow for timely adjustment of medications.

All pregnancies in transplant recipients should be considered high risk pregnancies and require close monitoring. Pregnant transplant recipients should be referred to an obstetrician as soon as pregnancy is confirmed.

Mycrophenolate derivatives (Mycophenolate mofetil /Cellcept, Mycophenolic acid /Myfortic) and mTOR inhibitors (Sirolimus /Rapamune, Everolimus /Certican) should be discontinued prior to pregnancy. If pregnancy occurs while on these medications they should be discontinued immediately and patients should be informed of the risks of teratogenicity.

ACE inhibitor use should be avoided, especially in the third trimester.

The recommended transplant follow up for pregnant transplant recipients is:

- Monthly for the first 28 weeks
- Every 2 weeks from week 28 to 32
- Weekly after week 32

At each visit special attention should be paid to:

- Rise in blood pressure
- New onset or significant increase of proteinuria
- Changes in serum creatinine
- Changes in immunosuppressive drug levels

The following are potential risks of pregnancy in transplant recipients:

- Increased incidence of first trimester miscarriages.
- Increased incidence of pre-eclampsia characterized by hypertension, edema, and proteinuria. Pre-eclampsia should be aggressively treated with hospitalization and urgent delivery if patients do not respond to more conservative means.
- Increased incidence of premature and low birth weight infants.
- Increased incidence of congenital malformation.

Postpartum

Postpartum monitoring of the transplant recipient is essential. Immunosuppressive drug levels should be monitored due to changing gastrointestinal function and absorption, loss of effects of fetal liver metabolism, reconstitution of the maternal immune system, the potential for postpartum depression and medication nonadherence.

The recommended management plan:

- Monitor immunosuppressive drug levels and alter doses and regimen for at least 1 month postpartum specially if doses increased during pregnancy
- Begin contraception when appropriate
- Mental health counseling if needed for postpartum depression

**Breast feeding:**

Counsel pregnant recipients, and their partners, about the risks and benefits of breast feeding. Most Immunosuppressive medication pass through the milk however, the risk to infant is not known. Combining breast feeding with bottle feeding may be a reasonable option. Breastfeeding mothers may consider monitoring blood levels of tacrolimus if toxicity is suspected.

**CHAPTER VI**

**MINIMIZATION AND PROTOCOL BIOPSIES**

**Chapter VI. Minimization and Protocol Biopsies**

- **Steroid**
  1. Steroid withdrawal may be considered for pediatric recipients and patients who develop NODAT (PTDM)
  2. Avoid steroid withdrawal in the following circumstances:

1. Highly sensitized, HLA or ABO incompatible transplants
2. Patients with previous rejection episodes

- **Tacrolimus**

Minimize the dose or convert to sirolimus (≥ 3 – 6 months post engraftment)

**Indications:**

- - - IF, TA, FIH and CNI toxicity
    - Kaposi sarcoma
    - BKV nephropathy
- **MMF**

Minimize or discontinue MMF in BKV nephropathy

**Protocol Biopsy**

**Schedule**

**12**

**6**

**0**

**Months post engraftment**

**Protocol Biopsy**

**Indications:**

1. HLA incompatible transplant
2. ABO incompatible transplant

**CHAPTER VII**

**MONITORING AND MANAGEMENT OF**

**DE NOVO HLA DSA**

**Chapter VII. Monitoring and Management of De Novo HLA DSA**

**Monitoring for de novo HLA DSA is individualized and targeted for patients at risk as follows:**

**Patients at risk for de novo HLA DSA*:**

1. Patients with late ACR (≥6 months post engraftment)
2. Patients with history of non-adherence to IS
3. Following physician-guided minimization of IS
4. Patients with significant HLA class II mismatches (≥3 MM at DR and DQ loci)

**Methodology of monitoring:**

- Monitoring will be performed at six month intervals from the second year post engraftment or at the time of late rejection as defined.
- Sera will be screened initially for HLA antibodies and if positive, single antigen class I and/or II will be performed
- Once de novo DSA is detected, closer surveillance is indicated (more frequent clinic visits and or low-threshold for protocol and indication biopsy)

**Schedule for protocol biopsy:**

**0**

**6**

**Months post DSA detection**

**1**

**2**

**3**

**4**

**Years post DSA detection**

**Subclinical AMR should be treated like clinical AMR*

**CHAPTER VIII**

**MONITORING AND MANAGEMENT OF BKV NEPHROPATHY**

**Chapter VIII. Monitoring and Management of BKV Nephropathy**

**Monitoring for BKV nephropathy**

- Maintain a high index of clinical suspicion in patients at risk.
- Low threshold for indication biopsy
- All biopsies with ACR beyond 3 months of engraftment will be stained for SV40

**Management of BKV nephropathy**

All patients with established BKV nephropathy will be subjected to the following management guidelines:

- 1. Discontinuation of MMF
  2. IVIG 1 – 2 gm/kg
  3. Minimization of Tacrolimus dose as per physician discretion
  4. Periodic monitoring of quantitative BKV PCR until it becomes negative

**CHAPTER IX**

**MANAGEMENT OF HLA AND ABO INCOMPATIBLE TRANSPLANTATION**

**Chapter IX. Management of HLA and ABO Incompatible Transplantation**

1. **HLA incompatible LD kidney transplantation**

Sensitized LD recipients with positive crossmatch (CXM) are candidates for desensitization. The desensitization regimen is individualized according to immune risk stratification as follows:

**High risk:**

- - 1. Positive T IgG CXM by AHG CDC and PD flow CXM (class I HLA antibodies), and/or positive B IgG CXM by CDC and/or PD flow CXM (class I and/or class II HLA antibodies);
    2. Positive DSA (class I and/or class II HLA antibodies) by solid phase assay

High risk patients as defined receive the following preconditioning regimen:

- Anti CD 20 antibody (Rituximab^®^): single dose of 500 mg IV over 10 hours, preferably two weeks prior to transplant.
- Immunoadsorption (IA) or plasma exchange (TPE), followed by small dose IVIG (100 mg/kg)
- IV prophylactic wide spectrum antibiotic (e.g. Cefoxitin) during IA or TPE
- The remaining of high dose IVIG (2gm/kg) over 48 hours prior to the day of surgery. (*Please refer to IVIG Guidelines*)

**Target prior to transplantation: negative T cell IgG AHG CDC CXM**

**and/or negative B cell CDC CXM**

**Intermediate risk:**

1. Negative T IgG and B IgG CXM by AHG CDC and CDC;
2. Positive T and/or B IgG by PD flow CXM;
3. Positive DSA (class I and/or class II HLA antibodies) by solid phase assay

Moderate risk patients receive the following preconditioning regimen:

- Anti CD 20 antibody (Rituximab^®^): single dose of 500 mg IV over 10 hours, preferably two weeks prior to transplant.
- High dose IVIG (2gm/kg) over 48 hours (*Please refer to IVIG Guidelines*)

**Relative Low risk:**

1. Negative T IgG and B IgG CXM by CDC and AHG CDC;
2. Negative T and B IgG by PD flow CXM;
3. Positive DSA (class I and/or class II HLA antibodies) by solid phase assay (MFI: 1000 – 2000)

Low risk patients receive the following preconditioning regimen:

- - Small dose IVIG (1 gm/kg) over 12-24 hours

1. **ABO incompatible LD kidney transplantation**

Desensitization regimen is similar to desensitization of high risk HLA incompatible transplantation with the exception of the timing of the infusion of the remaining dose of high dose IVIG. The dose is administered at the conclusion of the first two weeks post engraftment.

**Target: Room temperature Isoagglutinin titer of ≤ 4 at the time of transplantation.**

**CHAPTER X**

**ADULT VACCINATION SCHEDULE**

**Chapter X. Adult Vaccination Schedule**

**Adult Vaccination Schedule for Kidney Transplantation**

| **Recommended inactivated vaccine** | **Pre-transplant** | | **Post-transplant** |
| --- | --- | --- | --- |
| **No-live vaccines** |  | |  |
| Pneumococcal  Polysaccharide (a 23-valent conjugated 7-, 10-, or 13- valent) | Indicated – transplant candidates should all receive polysaccharide vaccine | | Indicated – 23 valent polysaccharide vaccine is recommended. One booster after 3 – 5 years |
| Trivalent inactivated influenza vaccine | Indicated annually | | Indicated annually |
| Hepatitis B | Indicated –accelerated schedule should be considered to complete series prior to transplant | Indicated – if not administered pre-transplant | |
| Hepatitis A | Indication for all patients | Indicated – give primary dosages if not done prior to transplant | |
| Meninggococcus:  Conjugated (MCV4) or Polysaccharide (MPSV4) | Indicated for individuals at risk as per general guidelines. Should receive MCV4 | Indicated – if not previously received, high risk individuals should receive MCA4 as per general guidelines | |
| Human papillomavirus (HPV): Quadrivalent (types 6, 11, 16, 18) or  Bivalent adjuvanted (types 16, 18) | Indicated for individuals as per general guidelines | Indicated as per general guidelines if not previously administered | |
| **Live vaccines** |  |  | |
| Varicella | Indicated.  Immunize all seronegative candidates at least 1 month prior to transplant | Not recommended | |

**CHAPTER XI**

**SCORE POINT SYSTEM FOR ALLOCATION OF DECEASED DONOR KIDNEY**

**Chapter XI. Score Point System for Allocation of Deceased Donor Kidney**

| FACTOR | POINTS | CONDITION |
| --- | --- | --- |
| Time waiting | 0.1 for each month of waiting time |  |
| HLA MM  (DR, DQ) | 1 point for each match at DR or DQ loci | Total of 4 points for zero HLA MM at DR and DQ loci |
| Virtual crossmatch (CXM) compatible sensitized patients | 4 points | Virtual CXM compatible candidates confirmed by final CXM |
| Kidney donor | 4 points | Kidney donor who develops ESRD |

**Thymoglobulin^®^**

**Guidelines for Use at KFSH&RC for Induction in Renal Transplant Patients**

**T**hymoglobulin^®^ is a purified, pasteurized preparation of gamma immune globulin (IgG] obtained from rabbits immunized with human thymocytes. It consists of cytotoxic polyclonal antibodies directed against antigens expressed on human T-lymphocytes. The manufacturing process includes the use of human erythrocytes to deplete cross-reactive antibodies to non-T-cell antigens. Also, potential exogenous viruses are removed or inactivated.

|  | - **Nursing Implications:** |
| --- | --- |

- In patients who will take rituximab (Mabthera®), always make sure that you infuse thymoglobulin before you start rituximab.
- Always remember to give premedications and use in-line filter (See dosage and administration)
- Do not infuse thymoglobulin thru the Y-site with any other solution except NS without checking first with the pharmacy.

|  | - **Dosage and Administration** |
| --- | --- |

- Induction: 1.5 mg /kg/dose (actual body weight) (round to the nearest 25 mg) Before each dose, the patient may be pretreated with diphenhydramine 25-50 mg PO/IV and acetaminophen 650 mg PO 1 hr prior infusion.
- Thymoglobulin^®^ should be given after the scheduled methylprednisolone dose
- Thymoglobulin^®^ dose must be diluted in normal saline (usually 250 mL) to a concentration not exceeding 0.5 mg/mL.
- 1^st^ dose should be infused over a minimum of 6 hrs into a **central line** through an in-line 0.22 µm filter. Subsequent doses can be given over 4 hrs.
- Peripheral administration: Thymoglobulin should always be given thru a central line, however, peripheral administration has been used in Europe (not approved in the USA). For peripheral administration thymoglobulin should be diluted in 500 cc NS, heparin 1000 units and hydrocortisone (20-100 mg) are added to the bag
- Infusion should be stopped immediately if a systemic reaction (dyspnea, tachycardia, hypotension, or a generalized skin reaction) or anaphylaxis occurs.
- Anaphylaxis has been reported with Thymoglobulin use in rare cases. In such cases, the infusion should be terminated immediately. Emergency treatment [0.3 mL to 0.5 mL of 1:1000 epinephrine SQ, corticosteroids, iv fluids, pressor amines, etc. should be provided as clinically indicated. Thymoglobulin or other rabbit immunoglobulins should not be administered again for such patients.

| 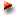 | **Monitoring** |
| --- | --- |

- In patients with immediate graft function, the first three doses will be given in 3 consecutive days automatically. Monitor Absolute Lymphocyte Count (ALC), goal < 200. On post-op day +3, if ALC > 200, order CD3 count (goal< 20 cells/mm^3^) or CD2 count (goal < 50 cell/mm^3^).
- In patients with delayed graft function (DGF), 3 doses will be given consecutively without the need to check CD3 count; however, automated absolute lymphocyte count (ALC) will be checked daily. After the 3^rd^ dose, if ALC > 200, check CD3 count (goal< 20 cells/mm^3^) or CD2 count (goal < 50 cell/mm^3^) and dose according to the following:
  - If CD3 ≤ 20 or CD2 ≤50, next dose can be skipped
  - If CD3 > 20 or CD2 >50, give another dose; check CD3 every other day thereafter and follow same guidelines.
- The dose should be reduced or discontinued in the presence of severe thrombocytopenia or leukopenia, according to the following guidelines:

| **Thymoglobulin Dosage Guideline (full dose = 1.5 mg/kg)** | | |
| --- | --- | --- |
| **WBC** | **Thymo Dose** | **Platelets** |
| > 3000 | Full dose | >75, 000 |
| 2000 - 3000 | ½ dose | 50,000 - 75,000 |
| <2000 | none | <50,000 |

**Alemtuzumab (Campath-1H)**

**Guidelines for Use at KFSH&RC for Induction in Renal Transplant Patients**

Note:

- - Campath is not part of KFSHRC induction or treatment of rejection protocol. The guidelines below are included only in case campath is to be used outside the protocol and after agreement of all transplant tream members. Also, only campath-1H^®^ brand will be used and not Lemtrada^®^

Inclusion criteria:

- Age 18-60 years of age with any degree of HLA mismatch
- No pre-transplant donor-specific antibody (DSA)
- Negative T cell and B cell crossmatch
- Thymoglobulin maximum dose mg/kg use in previous transplant(s)

Exclusion criteria:

- ABO incompatible
- HLA identical or zero mismatched donor
- HBV, HCV, HIV
- History of malignancy in the past 5 years
- Simultaneous pancreas-kidney transplantation (SPK)

Dosing/administration:

- SC administration (lower infusion-related reactions): administer 30 mg pre-operatively as a slow (1 minute) SC injection. This should be done at 7 am in the floor before patient is transferred to OR
- Pre-medications: diphenhydramine 50 mg, acetaminophen 500-1000 mg (30 minutes before infusion)
- Methylprednisolone 30-60 minutes before Alemtuzumab dose

Monitoring parameters:

- Vital signs; prior to and during infusions
- CBC with differential; prior to and post treatment then monthly thereafter until lymphopenia resolve
- Thyroid function (thyroid stimulating hormone level); prior to treatment then annually for 48 months post-treatment
- Donor-specific antibodies (DSA); prior to treatment then annually for at least 48 months post-treatment
- BK and CMV; monthly then every 3 months

**Intravenous Immune Globulin Use in Renal Transplant Recipients**

Approved by MCA, Executive Director, 18 March 2007

1. **Generic and Brand Name of Drug:**

Octagam^®^ (or any glucose/sucrose free product)

1. **Reason(s) for Guidelines**
   1. Expensive medication
   2. Waiver of form B requirement
   3. Inclusion of IVIG use in renal transplant recipients in the IVIG indication form
2. **Prescribing Restrictions:**

At KFSH&RC, accepted uses include treatment of primary immunodeficient diseases, select postallogeneic BMT patients, immune mediated thrombocytopenias, Kawasaki disease, and select HIV infected children. (See IVIG indication form)

1. **Indication(s) for which use of drug at KFSH & RC is requested, labeling status, dose, and age group.**
   1. As part of a desensitization protocol applied for highly sensitized adult renal transplant candidates who have a positive cross match test with their prospective donors. Level of evidence Ib, randomized controlled trials (see attached). This is an unapproved indication (see dose below).
   2. In combination with other modalities for the treatment of acute antibody mediated rejection post renal transplantation in adult and pediatrics recipients. Level of evidence III, comparative and case control studies (see attached). This is an unapproved indication (see dose below).
   3. Steroid resistant acute cellular rejection in adults and children who fail or have a contraindication to polyclonal antibodies. Level of evidence Ib (see attached). This is an unapproved indication (see dose below).
2. **Inclusion criteria**
   1. Desensitization protocols (Indication number1)
      1. Potential adult renal transplant recipients who have a positive flow cytometry cross match test in the presence of donor specific antibodies with a negative AHG-CDC crossmatch. Used in combination with other modalities as part of a preconditioning regimen.
      2. Potential adult renal transplant recipients who have a positive flow cytometry and AHG-CDC cross match test. Used in combination with other modalities as part of a preconditioning regimen.
   2. Antibody mediated rejection (Indication number 2)

Adult and pediatric renal transplant recipients who develop acute antibody mediated rejection (AMR) characterized by the presence of two of the following criteria: acute allograft dysfunction, pathological findings of AMR and/or the presence of donor specific anti-HLA antibodies used alone or in combination with immunoadsorption or total plasma exchange.

- 1. Steroid resistant rejection (Indication number 3)

Adult and pediatric renal transplant recipients with steroid resistant acute cellular rejection who fail, or have a contraindication to polyclonal antilymphocytes antibodies.

1. **Exclusion criteria**
2. Adult and pediatric renal transplant recipients who have chronic antibody mediated rejection.
3. First line therapy for acute cellular rejection in adult and pediatric renal transplant recipients.
4. Hypersensitivity to immune globulin or any component of the formulation.
5. Patients known to have selective IgA deficiency.
6. **Dose, Frequency, Route, Rate, and Duration of Therapy**
7. Desensitization protocol: 2 gm/kg (MAXIMUM 140 GM) IV infusion over 48 hours. Premedicate with acetaminophen 650 mg P.O. and diphenhydramine 25-50 mg IV, 30 minutes prior to infusion.
8. Treatment of acute antibody rejection: a total of 2 gm/kg given as 100 mg/kg after each plasmapheresis session, then to give the rest of 2 gm/kg over 24 hours after the last session of plasmapheresis.
9. Treatment of steroid resistant rejection: 500 mg/kg/day for 7 days
10. **Common Adverse Reactions**

Cardiovascular: Flushing of the face, tachycardia, hyper-/hypotention, chest tightness, angioedema, lightheadedness, chest pain, MI, CHF, pulmonary embolism

Central nervous system: Anxiety, chills, dizziness, drowsiness, fatigue, fever, headache, irritability, lethargy, malaise, aseptic meningitis syndrome

Dermatologic: Pruritus, rash, urticaria

Gastrointestinal: Abdominal cramps, diarrhea, nausea, sore throat, vomiting

Hematologic: Autoimmune hemolytic anemia, mild hemolysis

Local: Pain or irritation at the infusion site

Neuromuscular & skeletal: Arthralgia, back or hip pain, myalgia, nuchal rigidity

Renal: Acute renal failure, acute tubular necrosis, anuria, BUN increased, creatinine increased, nephrotic syndrome, oliguria, proximal tubular nephropathy, osmotic nephrosis

Respiratory: Cough, dyspnea, wheezing, nasal congestion, rhinorrhea, sinusitis

Miscellaneous: Diaphoresis, hypersensitivity reactions, anaphylaxis

1. **Rare/Serious Adverse Reactions**

Apnea, ARDS, bronchospasm, bullous dermatitis, cardiac arrest, Coombs’ test positive, cyanosis, epidermolysis, erythema multiforme, hepatic dysfunction, hypoxemia, leukopenia, loss of consciousness, pancytopenia, pulmonary edema, rigors, seizure, Stevens-Johnson syndrome, thromboembolism, transfusion-related acute lung injury (TRALI), tremor, vascular collapse

1. **Drug Interactions**

Live virus, vaccines (e.g., measles, mumps, rubella): May have impaired response to vaccines; separate administration by at least 3 months

1. **Monitoring Parameters**

Renal function, urine output, hemoglobin and hematocrit, infusion-related adverse reactions, and anaphylaxis
