# Supplementary material for: Standard induction with basiliximab versus no induction in low immunological risk kidney transplant recipients: study protocol for a randomized controlled trial
Source: Trials. 2021 Jun 24;22:414. doi: 10.1186/s13063-021-05253-1 (PMC8223264; doi:10.1186/s13063-021-05253-1)
Supplement: Supplementary file 2 [file 13063_2021_5253_MOESM2_ESM.docx]

this study.

LENGTH OF STUDY

You will be followed for 1 year after your transplant as part of this study. Also, you will be followed up in KFSHRC indefinitely after transplant as a usual care.

A. Potential Risks and Discomforts:

If you choose to take part in this study, there is a risk that:

- Opportunistic infections: The incidence of opportunistic infections may be increased by immunosuppressive therapy.
- Lose time at work or home and spend more time in the hospital or doctor's office than usual

D. Potential Benefits:

It is not possible to know at this time if not taking the drug is better than the usual approach so this study may or may not help you. This study will Help researchers learn new thing that will help people in the future

E. Alternative to Participation (where applicable):

I will continue to receive the required clinical care with or without participating in the study

F. Cost/s Reimbursements:

There is no cost of treating your condition in this study. All the drugs are part of standard of care and will be provided by KFSH&RC.

مدة الدراسة:

ستخضع للمتابعة لمدة ١ سنة بعد الزرع كجزء من هذه الدراسة. وسيتم متابعتك في مستشفى الملك فيصل التخصصي ومركز الأبحاث إلى اجل غير مسمى بعد الزرع كالعناية المعتادة

جـ المخاطر والانزعاجات المحتملة:

إذا اخترت المشاركة في هذه الدراسة ، فهناك خطر من

- الالتهابات الانتهازية:سيكون هناك خطر زيادة حالات العدوى الانتهازية عن طريق العلاج لتثبيط المناعة.
- ستقوم بقضاءلوقت إضافي في المستشفى أو مكتب الطبيب أكثر من المعتاد مما يؤدي الى تقليل الساعات التي تقضيها في العمل أو المنزل.

د. الفوائد المحتملة:

ليس من الممكن أن تعرف في هذا الوقت ما إذا كان تناول الدواء افضل من الطريقة المعتادة لذلك قد تساعدك هذه الدراسة أو لا تساعدك. ستساعد هذه الدراسة الباحثين على تعلم شيء جديد من شأنه مساعدة الناس في المستقبل

هـ البدائل عن المشاركة (إن وجدت):

البديل هو عدم المشاركة في هذه الدراسة البحثية وفي هذه الحالة ستستمر
ر في تلقي الرعاية السريرية المطلوبة .

و. التكاليف / التعويضات المالية:

لا يوجد أي تكلفة لعلاج حالتك في هذه الدراسة. جميع الأدوية هي جزء من مستوى الرعاية وسيتم توفيرها من قبل مستشفى الملك فيصل التخصصي ومركز الأبحاث. كما لن يتم منحك مقابل مالي جراء مشاركتك في الدراسة.
